# *catena*-Poly[[[diaquacadmium(II)]bis[μ-2-(pyridinium-1-yl)butanedioato]-κ^2^ *O* ^1^:*O* ^4^;κ^2^ *O* ^4^:*O* ^1^] tetrahydrate], a polymeric chain structure

**DOI:** 10.1107/S1600536810041607

**Published:** 2010-10-23

**Authors:** Zhi-Hua Liu, Sen Zhu

**Affiliations:** a Department of Materials Science and Engineering, Tianjin Institute of Urban Construction, Tianjin 300384, People’s Republic of China

## Abstract

In the title complex, {[Cd(C_9_H_8_NO_4_)_2_(H_2_O)_2_]·4H_2_O}_*n*_, the Cd^II^ atom (site symmetry 2) is coordinated by six O atoms from four crystallographically related 1-(1,2-dicarboxyl­ate)pyridin-1-ium ligands (*L*) and from two water molecules in a distorted octahedral geometry. Paired *L* ligands connect Cd^II^ atoms into a chain motif parallel to [001], which is further inter­linked by O—H⋯O hydrogen bonds into a three-dimensional supra­molecular net.

## Related literature

For ligands including pyridyl and carboxyl­ate groups as building tectons of the supra­molecular lattice in inorganic–organic coordination chemistry, see: Batten (2001 ▶); Kitagawa & Matsuda (2007 ▶). 
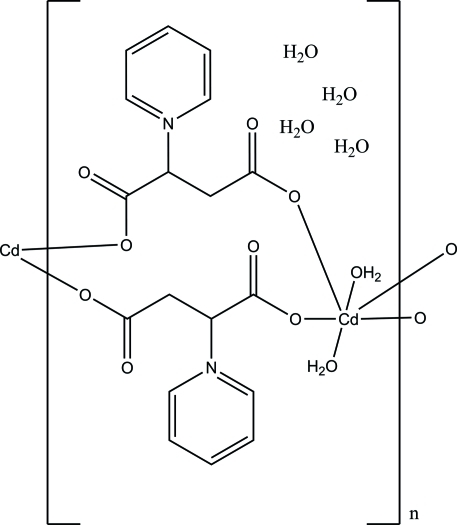


## Experimental

### 

#### Crystal data

[Cd(C_9_H_8_NO_4_)_2_(H_2_O)_2_]·4H_2_O
*M* *_r_* = 608.82
Monoclinic, 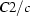

*a* = 17.612 (4) Å
*b* = 9.798 (2) Å
*c* = 14.076 (3) Å
β = 102.63 (3)°
*V* = 2370.2 (8) Å^3^
*Z* = 4
Mo *K*α radiation
μ = 1.00 mm^−1^
*T* = 294 K
0.28 × 0.22 × 0.20 mm

#### Data collection

Bruker SMART APEX CCD area-detector diffractometer
Absorption correction: multi-scan (*SADABS*; Sheldrick, 1996 ▶) *T*_min_ = 0.768, *T*_max_ = 0.826
2598 measured reflections
2086 independent reflections
1854 reflections with *I* > 2σ(*I*)
*R*_int_ = 0.026

#### Refinement

*R*[*F* ^2^ > 2σ(*F* ^2^)] = 0.036
*wR*(*F* ^2^) = 0.092
*S* = 1.14
2086 reflections
159 parameters
H-atom parameters constrained
Δρ_max_ = 0.75 e Å^−3^
Δρ_min_ = −0.53 e Å^−3^

### 

Data collection: *SMART* (Bruker, 2003 ▶); cell refinement: *SAINT* (Bruker, 2003 ▶); data reduction: *SAINT*; program(s) used to solve structure: *SHELXS97* (Sheldrick, 2008 ▶); program(s) used to refine structure: *SHELXL97* (Sheldrick, 2008 ▶); molecular graphics: *DIAMOND* (Brandenburg, 2005 ▶); software used to prepare material for publication: *SHELXTL* (Sheldrick, 2008 ▶).

## Supplementary Material

Crystal structure: contains datablocks I, global. DOI: 10.1107/S1600536810041607/kj2155sup1.cif

Structure factors: contains datablocks I. DOI: 10.1107/S1600536810041607/kj2155Isup2.hkl

Additional supplementary materials:  crystallographic information; 3D view; checkCIF report

## Figures and Tables

**Table 1:** Selected bond lengths (Å)

Cd1—O1	2.271 (3)
Cd1—O1^i^	2.271 (3)
Cd1—O5^i^	2.284 (3)
Cd1—O5	2.284 (3)
Cd1—O3^ii^	2.298 (3)
Cd1—O3^iii^	2.298 (3)

Symmetry codes: (i) 
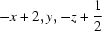
; (ii) 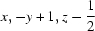; (iii) 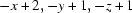.

**Table 2:** Hydrogen-bond geometry (Å, °)

*D*—H⋯*A*	*D*—H	H⋯*A*	*D*⋯*A*	*D*—H⋯*A*
O5—H5*A*⋯O4^iii^	0.85	1.83	2.649 (6)	162
O5—H5*B*⋯O7^iv^	0.85	1.91	2.680 (5)	150
O6—H6*A*⋯O7^v^	0.85	2.24	2.964 (6)	143
O6—H6*B*⋯O2^vi^	0.85	1.90	2.753 (5)	177
O7—H7*A*⋯O6^vii^	0.84	1.93	2.753 (6)	169
O7—H7*B*⋯O4^viii^	0.90	1.80	2.700 (5)	172

Symmetry codes: (iii) 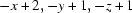; (iv) 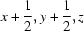; (v) 
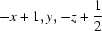
; (vi) 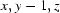; (vii) 
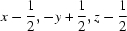
; (viii) 
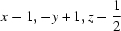
.

**Fig. 1.**
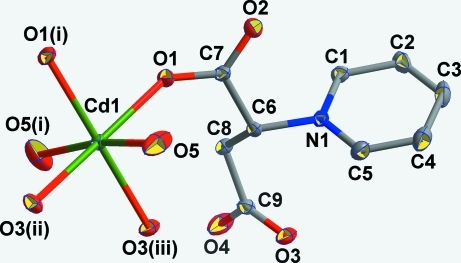
The molecular structure with atom-labelling scheme and ellipsoids drawn at the 50% probability level. Symmetry operations: (i) -x+2, y, -z+1/2; (ii) x, -y+1, z-1/2; (iii) -x+2, -y+1, -z+1.

**Fig. 2.**
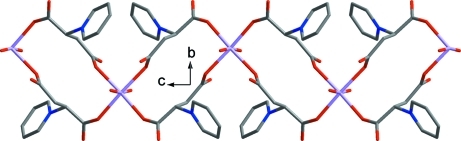
View of the one-dimensional polymeric chain along the [001] direction.

**Fig. 3.**
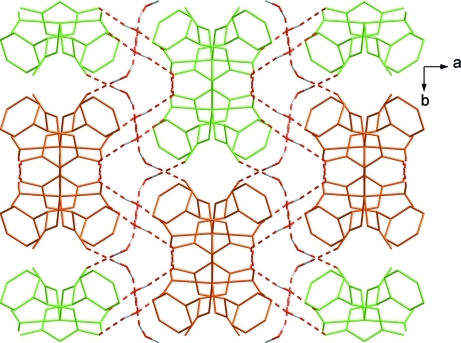
Part of the three-dimensional supramolecular net, showing the hydrogen bonds in red dashed lines. H atoms not involved in H-bonsing have been omitted for clarity.

## supplementary crystallographic information

### Comment

Versatile ligands involving pyridyl and carboxylate groups have been proven to be effective building tectons of supramolecular lattice in the field of inorganic-organic coordination chemistry (Batten, 2001; Kitagawa & Matsuda, 2007).

In this paper, 1-(1,2-dicarboxyethyl)pyridin-1-ium chloride was employed as a bridging ligand to assemble with Cd^II^ into a one-dimensional polymeric chain motif, in which the coordination geometry of Cd^II^ can be portrayed as a distorted octahedron (CdO_6_) (Fig. 1). With the aid of the two monodentate carboxylates of *L*, the adjacent Cd^II^ ions are further interlinked to afford a chain motif along the [001] direction (Fig. 2). Additionally, strong O—H···O bonds are found between the coordinated water ligands, carboxylates, and lattice water molecules, to generating a complicated three-dimensional supramoleculecular lattice (Fig. 3).

### Experimental

A water solution (8 ml) containing CdCl_2_(18.4 mg, 0.1 mmol) and 1-(1,2-dicarboxyethyl)pyridin-1-ium chloride (23.1 mg, 0.1 mmol) was heated to 373 K for 24 h and subsequently cooled to room temperature at a rate of 1 K/h. Colorless block shape crystals were obtained.

### Refinement

All H atoms were initially located in a difference Fourier map. The C—H atoms were then constrained to an ideal geometry, with C—H distance of 0.93 Å, and *U*_iso_(H) = 1.2*U*_eq_(C). The O-bound hydrogen atoms were first located in difference Fourier maps, and then fixed in calculated sites, with d(O—H) = 0.84–0.90Å.

### Crystal data

**Table:**

[Cd(C_9_H_8_NO_4_)_2_(H_2_O)_2_]·4H_2_O	*F*(000) = 1240
*M**_r_* = 608.82	*D*_x_ = 1.706 Mg m^−^^3^
Monoclinic, *C*2/*c*	Mo *K*α radiation, λ = 0.71073 Å
*a* = 17.612 (4) Å	Cell parameters from 2290 reflections
*b* = 9.798 (2) Å	θ = 2.5–22.0°
*c* = 14.076 (3) Å	µ = 1.00 mm^−^^1^
β = 102.63 (3)°	*T* = 294 K
*V* = 2370.2 (8) Å^3^	Block, colourless
*Z* = 4	0.28 × 0.22 × 0.20 mm

### Data collection

**Table:**

Bruker SMART APEX CCD area-detector diffractometer	2086 independent reflections
Radiation source: fine-focus sealed tube	1854 reflections with *I* > 2σ(*I*)
graphite	*R*_int_ = 0.026
phi and ω scans	θ_max_ = 25.0°, θ_min_ = 2.4°
Absorption correction: multi-scan (*SADABS*; Sheldrick, 1996)	*h* = −1→20
*T*_min_ = 0.768, *T*_max_ = 0.826	*k* = −1→11
2598 measured reflections	*l* = −16→16

### Refinement

**Table:**

Refinement on *F*^2^	Primary atom site location: structure-invariant direct methods
Least-squares matrix: full	Secondary atom site location: difference Fourier map
*R*[*F*^2^ > 2σ(*F*^2^)] = 0.036	Hydrogen site location: inferred from neighbouring sites
*wR*(*F*^2^) = 0.092	H-atom parameters constrained
*S* = 1.14	*w* = 1/[σ^2^(*F*_o_^2^) + (0.0445*P*)^2^ + 4.7881*P*] where *P* = (*F*_o_^2^ + 2*F*_c_^2^)/3
2086 reflections	(Δ/σ)_max_ < 0.001
159 parameters	Δρ_max_ = 0.75 e Å^−^^3^
0 restraints	Δρ_min_ = −0.53 e Å^−^^3^

### Special details

**Table:**

Geometry. All e.s.d.'s (except the e.s.d. in the dihedral angle between two l.s. planes) are estimated using the full covariance matrix. The cell e.s.d.'s are taken into account individually in the estimation of e.s.d.'s in distances, angles and torsion angles; correlations between e.s.d.'s in cell parameters are only used when they are defined by crystal symmetry. An approximate (isotropic) treatment of cell e.s.d.'s is used for estimating e.s.d.'s involving l.s. planes.
Refinement. Refinement of *F*^2^ against ALL reflections. The weighted *R*-factor *wR* and goodness of fit *S* are based on *F*^2^, conventional *R*-factors *R* are based on *F*, with *F* set to zero for negative *F*^2^. The threshold expression of *F*^2^ > σ(*F*^2^) is used only for calculating *R*-factors(gt) *etc*. and is not relevant to the choice of reflections for refinement. *R*-factors based on *F*^2^ are statistically about twice as large as those based on *F*, and *R*- factors based on ALL data will be even larger.

### Fractional atomic coordinates and isotropic or equivalent isotropic displacement parameters (Å^2^)

**Table:**

	*x*	*y*	*z*	*U*_iso_*/*U*_eq_	
Cd1	1.0000	0.64349 (4)	0.2500	0.02528 (15)	
O1	0.9894 (2)	0.8142 (3)	0.3555 (2)	0.0422 (8)	
O2	0.9118 (2)	0.9428 (3)	0.4220 (2)	0.0462 (8)	
O3	1.00096 (16)	0.5232 (3)	0.6354 (2)	0.0341 (7)	
O4	1.12761 (18)	0.5582 (4)	0.6576 (3)	0.0524 (9)	
O5	0.8676 (2)	0.6277 (4)	0.2046 (3)	0.0687 (12)	
H5A	0.8626	0.5792	0.2530	0.103*	
H5B	0.8432	0.7031	0.1969	0.103*	
O6	0.7816 (2)	0.0490 (4)	0.4730 (3)	0.0579 (10)	
H6A	0.7535	0.0987	0.4297	0.087*	
H6B	0.8219	0.0142	0.4590	0.087*	
O7	0.2566 (2)	0.3103 (4)	0.1327 (3)	0.0616 (11)	
H7A	0.2647	0.3431	0.0808	0.092*	
H7B	0.2122	0.3469	0.1436	0.092*	
N1	0.91334 (18)	0.7734 (3)	0.5733 (2)	0.0239 (7)	
C1	0.9343 (2)	0.8872 (4)	0.6275 (3)	0.0280 (9)	
H1	0.9779	0.9367	0.6209	0.034*	
C2	0.8904 (3)	0.9292 (5)	0.6926 (3)	0.0365 (10)	
H2	0.9051	1.0062	0.7308	0.044*	
C3	0.8258 (3)	0.8577 (6)	0.7007 (3)	0.0471 (12)	
H3	0.7957	0.8865	0.7437	0.057*	
C4	0.8052 (3)	0.7424 (6)	0.6447 (3)	0.0466 (12)	
H4	0.7612	0.6927	0.6497	0.056*	
C5	0.8502 (3)	0.7015 (5)	0.5813 (3)	0.0349 (10)	
H5	0.8368	0.6234	0.5438	0.042*	
C6	0.9595 (2)	0.7326 (4)	0.5014 (3)	0.0255 (8)	
H6	0.9377	0.6466	0.4718	0.031*	
C7	0.9515 (2)	0.8395 (4)	0.4189 (3)	0.0275 (9)	
C8	1.0448 (2)	0.7062 (4)	0.5496 (3)	0.0285 (9)	
H8A	1.0732	0.6894	0.4990	0.034*	
H8B	1.0661	0.7882	0.5840	0.034*	
C9	1.0585 (2)	0.5875 (4)	0.6204 (3)	0.0281 (9)	

### Atomic displacement parameters (Å^2^)

**Table:**

	*U*^11^	*U*^22^	*U*^33^	*U*^12^	*U*^13^	*U*^23^
Cd1	0.0303 (2)	0.0218 (2)	0.0267 (2)	0.000	0.01280 (16)	0.000
O1	0.079 (2)	0.0277 (16)	0.0296 (15)	0.0071 (15)	0.0322 (16)	0.0015 (12)
O2	0.068 (2)	0.0403 (19)	0.0370 (17)	0.0216 (17)	0.0266 (16)	0.0156 (15)
O3	0.0361 (16)	0.0282 (16)	0.0382 (16)	−0.0014 (13)	0.0082 (13)	0.0115 (13)
O4	0.0336 (18)	0.053 (2)	0.068 (2)	0.0012 (16)	0.0067 (16)	0.0288 (19)
O5	0.0371 (19)	0.071 (3)	0.097 (3)	0.0077 (19)	0.0120 (19)	0.046 (2)
O6	0.054 (2)	0.063 (2)	0.059 (2)	0.0183 (19)	0.0183 (17)	0.0063 (19)
O7	0.052 (2)	0.057 (2)	0.083 (3)	0.0200 (18)	0.031 (2)	0.030 (2)
N1	0.0297 (17)	0.0241 (17)	0.0187 (15)	−0.0018 (14)	0.0069 (13)	0.0010 (13)
C1	0.036 (2)	0.025 (2)	0.0237 (18)	−0.0027 (17)	0.0075 (16)	0.0009 (15)
C2	0.049 (3)	0.039 (2)	0.0236 (19)	0.008 (2)	0.0114 (18)	−0.0039 (19)
C3	0.046 (3)	0.066 (3)	0.037 (2)	0.009 (3)	0.025 (2)	0.005 (3)
C4	0.040 (3)	0.058 (3)	0.046 (3)	−0.002 (2)	0.019 (2)	0.010 (2)
C5	0.040 (2)	0.033 (2)	0.031 (2)	−0.007 (2)	0.0091 (18)	0.0010 (18)
C6	0.036 (2)	0.0190 (19)	0.0241 (19)	0.0006 (17)	0.0128 (16)	−0.0013 (15)
C7	0.041 (2)	0.022 (2)	0.0219 (18)	0.0005 (18)	0.0113 (16)	0.0026 (16)
C8	0.034 (2)	0.024 (2)	0.030 (2)	−0.0007 (18)	0.0127 (17)	0.0053 (17)
C9	0.038 (2)	0.0198 (19)	0.027 (2)	−0.0006 (18)	0.0087 (17)	−0.0016 (16)

### Geometric parameters (Å, °)

**Table:**

Cd1—O1	2.271 (3)	N1—C1	1.356 (5)
Cd1—O1^i^	2.271 (3)	N1—C6	1.485 (5)
Cd1—O5^i^	2.284 (3)	C1—C2	1.384 (5)
Cd1—O5	2.284 (3)	C1—H1	0.9300
Cd1—O3^ii^	2.298 (3)	C2—C3	1.363 (7)
Cd1—O3^iii^	2.298 (3)	C2—H2	0.9300
O1—C7	1.250 (5)	C3—C4	1.381 (7)
O2—C7	1.236 (5)	C3—H3	0.9300
O3—C9	1.250 (5)	C4—C5	1.376 (6)
O3—Cd1^iii^	2.298 (3)	C4—H4	0.9300
O4—C9	1.248 (5)	C5—H5	0.9300
O5—H5A	0.8501	C6—C8	1.528 (5)
O5—H5B	0.8500	C6—C7	1.548 (5)
O6—H6A	0.8500	C6—H6	0.9800
O6—H6B	0.8500	C8—C9	1.517 (6)
O7—H7A	0.8391	C8—H8A	0.9700
O7—H7B	0.9029	C8—H8B	0.9700
N1—C5	1.341 (5)		
			
O1—Cd1—O1^i^	85.13 (14)	C3—C2—H2	120.0
O1—Cd1—O5^i^	95.28 (13)	C1—C2—H2	120.0
O1^i^—Cd1—O5^i^	90.45 (15)	C2—C3—C4	119.5 (4)
O1—Cd1—O5	90.45 (15)	C2—C3—H3	120.3
O1^i^—Cd1—O5	95.28 (13)	C4—C3—H3	120.3
O5^i^—Cd1—O5	172.2 (2)	C5—C4—C3	119.5 (4)
O1—Cd1—O3^ii^	175.18 (12)	C5—C4—H4	120.3
O1^i^—Cd1—O3^ii^	92.87 (10)	C3—C4—H4	120.3
O5^i^—Cd1—O3^ii^	89.11 (12)	N1—C5—C4	120.5 (4)
O5—Cd1—O3^ii^	85.37 (14)	N1—C5—H5	119.7
O1—Cd1—O3^iii^	92.87 (10)	C4—C5—H5	119.7
O1^i^—Cd1—O3^iii^	175.18 (12)	N1—C6—C8	112.0 (3)
O5^i^—Cd1—O3^iii^	85.37 (14)	N1—C6—C7	110.8 (3)
O5—Cd1—O3^iii^	89.11 (12)	C8—C6—C7	111.5 (3)
O3^ii^—Cd1—O3^iii^	89.46 (15)	N1—C6—H6	107.4
C7—O1—Cd1	138.2 (3)	C8—C6—H6	107.4
C9—O3—Cd1^iii^	127.4 (3)	C7—C6—H6	107.4
Cd1—O5—H5A	95.3	O2—C7—O1	125.6 (4)
Cd1—O5—H5B	115.7	O2—C7—C6	119.1 (3)
H5A—O5—H5B	116.7	O1—C7—C6	115.2 (3)
H6A—O6—H6B	116.6	C9—C8—C6	114.9 (3)
H7A—O7—H7B	108.3	C9—C8—H8A	108.5
C5—N1—C1	120.9 (3)	C6—C8—H8A	108.5
C5—N1—C6	120.2 (3)	C9—C8—H8B	108.5
C1—N1—C6	118.8 (3)	C6—C8—H8B	108.5
N1—C1—C2	119.6 (4)	H8A—C8—H8B	107.5
N1—C1—H1	120.2	O4—C9—O3	124.4 (4)
C2—C1—H1	120.2	O4—C9—C8	116.9 (4)
C3—C2—C1	120.0 (4)	O3—C9—C8	118.6 (4)
			
O1^i^—Cd1—O1—C7	141.1 (5)	C5—N1—C6—C7	111.7 (4)
O5^i^—Cd1—O1—C7	−128.9 (4)	C1—N1—C6—C7	−65.7 (4)
O5—Cd1—O1—C7	45.9 (4)	Cd1—O1—C7—O2	−126.2 (4)
O3^iii^—Cd1—O1—C7	−43.3 (4)	Cd1—O1—C7—C6	56.5 (6)
C5—N1—C1—C2	0.5 (6)	N1—C6—C7—O2	1.7 (5)
C6—N1—C1—C2	178.0 (3)	C8—C6—C7—O2	−123.8 (4)
N1—C1—C2—C3	−1.2 (6)	N1—C6—C7—O1	179.2 (3)
C1—C2—C3—C4	1.0 (7)	C8—C6—C7—O1	53.6 (5)
C2—C3—C4—C5	−0.1 (7)	N1—C6—C8—C9	64.2 (4)
C1—N1—C5—C4	0.3 (6)	C7—C6—C8—C9	−171.0 (3)
C6—N1—C5—C4	−177.1 (4)	Cd1^iii^—O3—C9—O4	10.1 (6)
C3—C4—C5—N1	−0.5 (7)	Cd1^iii^—O3—C9—C8	−172.2 (3)
C5—N1—C6—C8	−123.1 (4)	C6—C8—C9—O4	176.5 (4)
C1—N1—C6—C8	59.5 (4)	C6—C8—C9—O3	−1.3 (5)

Symmetry codes: (i) −*x*+2, *y*, −*z*+1/2; (ii) *x*, −*y*+1, *z*−1/2; (iii) −*x*+2, −*y*+1, −*z*+1.

### Hydrogen-bond geometry (Å, °)

**Table:**

*D*—H···*A*	*D*—H	H···*A*	*D*···*A*	*D*—H···*A*
O5—H5A···O4^iii^	0.85	1.83	2.649 (6)	162
O5—H5B···O7^iv^	0.85	1.91	2.680 (5)	150
O6—H6A···O7^v^	0.85	2.24	2.964 (6)	143
O6—H6B···O2^vi^	0.85	1.90	2.753 (5)	177
O7—H7A···O6^vii^	0.84	1.93	2.753 (6)	169
O7—H7B···O4^viii^	0.90	1.80	2.700 (5)	172

Symmetry codes: (iii) −*x*+2, −*y*+1, −*z*+1; (iv) *x*+1/2, *y*+1/2, *z*; (v) −*x*+1, *y*, −*z*+1/2; (vi) *x*, *y*−1, *z*; (vii) *x*−1/2, −*y*+1/2, *z*−1/2; (viii) *x*−1, −*y*+1, *z*−1/2.
